# Glucocorticoid receptor activity regulates light adaptation in the zebrafish retina

**DOI:** 10.3389/fncir.2013.00145

**Published:** 2013-09-24

**Authors:** Akira Muto, Michael R. Taylor, Miyuki Suzawa, Juan I. Korenbrot, Herwig Baier

**Affiliations:** Department of Physiology, University of California at San FranciscoSan Francisco, CA, USA

**Keywords:** glucocorticoid receptor, cortisol, vision, light adaptation, retina, optokinetic response, zebrafish, electroretinogram

## Abstract

Glucocorticoids modulate diverse aspects of physiology and behavior, including energy homeostasis, stress response, and memory, through activation of the glucocorticoid receptor (GR). Light perception has profound effects on the production of glucocorticoids via functional connections of the retina to the hypothalamus-pituitary-adrenal axis. We report here that glucocorticoids can also signal in the reverse direction, i. e., regulate visual function in zebrafish, *Danio rerio*. The zebrafish GR mutant, *gr*^*s357*^, harbors a missense mutation that completely blocks the transcriptional activity of GR. In this mutant, visual behavior was abolished following a period of darkness and recovered sluggishly after return to the light. Electrophysiological measurements showed that the photoresponse of the dark-adapted retina was reduced in the mutant and re-adapted to light with a substantial delay. Several gene products, including some that are important for dopaminergic signaling, were misregulated in *gr*^*s357*^ mutants. We suggest that GR controls a gene network required for visual adaptation in the zebrafish retina and potentially integrates neuroendocrine and sensory responses to environmental changes.

## INTRODUCTION

Glucocorticoids regulate diverse aspects of physiology, such as glucose metabolism, stress reactions, and neural plasticity (Kellendonk et al., 2002; Pecoraro et al., 2006; Krugers et al., 2012). In the HPA axis, the hypothalamus stimulates the pituitary to secrete adrenocorticotropic hormone (ACTH) into the bloodstream. ACTH acts on the adrenal gland (or its homolog the interrenal organ in teleosts), which synthesizes and secretes glucocorticoids (cortisol in fish and humans). Glucocorticoids in turn act broadly on peripheral tissues and the brain. Glucocorticoids provide negative feedback signals to the hypothalamus and the pituitary, thus terminating the stress response (Philips et al., 1997). Glucocorticoid levels in the blood oscillate in a circadian rhythm in all vertebrates, peaking at night or in the early morning and dropping in the afternoon (Baker and Rance, 1981).

The production of glucocorticoids is also regulated by light perception. Light signals are carried to the HPA axis through direct axonal connection of the retina to the hypothalamus. While this retinohypothalamic pathway has been extensively studied, the possibility that glucocorticoids may also reciprocally regulate visual function has not been explored. Recently, we identified a zebrafish glucocorticoid receptor (GR) mutant, *gr *^*s357*^ , in which transcriptional activity is abolished due to mutation of a single, highly conserved amino acid in the second zinc finger motif (Griffiths et al., 2012; Ziv et al., 2012). This mutant was originally discovered in a systematic screen with a set of behavioral assays, followed by morphological examinations. The mutant shows no apparent defects in the retinal histology or retinal ganglion cell axonal projection. In this mutant, visual background adaptation (VBA; namely, melanosome aggregation in response to light) is weakly affected, but the optokinetic response (OKR) and the optomotor response are comparable to wild type. Remarkably, the light-adaptation phenotype was discovered in a shelf-screen of VBA mutants in the OKR assay with a dark-to-light transition (Muto et al., 2005).

Here we have explored how light perception is affected by defective cortisol signaling in this mutant. We show using behavior and electrophysiology that visual adaptation of the retina is disrupted in *gr *^*s357*^ mutants. This direct route of communication from the HPA axis to the retina may help to integrate the function of the sensory periphery with global physiological states.

## MATERIALS AND METHODS

### MEASUREMENT OF THE OPTOKINETIC RESPONSE

The OKR assay was conducted as described previously (Muto et al., 2005). An animation of sine-wave gratings was projected on the internal wall of a drum (6 cm height, 5.6 cm in inner diameter), using an LCD projector (InFocus LP755, Portland, OR, USA). To focus the image at close distance, a wide-angle conversion lens (Kenko VC-050Hi, Tokyo, Japan), a close-up lens (King CU+1, Japan), and a neutral density filter (Hoya, ND4, Tokyo, Japan) were placed in front of the projector. Zebrafish larvae were immobilized in 2.5% methylcellulose in E3 egg water with their dorsal sides up in the inverted lid of a 3.5 cm diameter petri dish and placed into the center of the drum. The fish were imaged using a dissecting microscope (Nikon SMZ-800, Tokyo, Japan) and a CCD camera (Cohu MOD8215-1300, Poway, CA, USA) to observe horizontal eye movements. Sine-wave gratings with a spatial frequency of 20°/cycle moving at 10°/s were used. Image-J () was used for both stimulus generation and image analysis. Images were captured via an LG-3 video capture board (Scion Corp.) at 2 frames/s with Scion Java Package 1.0 for Image-J Windows. A custom-programmed Image-J plug-in (A. M., unpublished) was used to calculate the changes in eye angles.

The light-adaptation assay was carried out as described previously (Page-McCaw et al., 2004). Zebrafish larvae were placed in the dark for 45 min to allow dark-adaptation, then subjected to OKR recordings at 2, 8, 15, and 30 min after return to a bright environment (2,400 cd/m^2^ underneath the larvae; 400–600 cd/m^2^ at the internal drum wall, where the visual stimulus was projected).

### ELECTRORETINOGRAM RECORDING

The zebrafish larvae were anesthetized in 0.004% tricaine. Electroretinograms (ERGs) were recorded from dark- and light-adapted zebrafish larvae at 5–7 days post-fertilization (dpf) in response to 20 ms light flashes, as previously published (Page-McCaw et al., 2004). The corneal response was amplified by 10^3^, filtered at 0.1–100 Hz, and digitized. For the light stimulus, white light (81 mW/cm^2^ at log 0) was used with neutral density filters to obtain varying intensities.

To compare data among animals, the *b*-wave amplitude at each intensity (*bpeak(I)*) was normalized by dividing it by the maximum amplitude measured in the same fish (*bpeak*_max_). The peak amplitude dependence on light is well described by the Naka-Rushton function ${bpeak(I)/}_{{bpeak}_{\max}}={I/}_{I+\sigma }$, where *I* is intensity and σ is the flash intensity at half maximum amplitude. The variance in the value of *bpeak*_max_ is not biologically significant and arises because the ERG is measured on the eye surface and varies in amplitude with electrode position and the extent to which individual larvae are submerged in the recording solution.

To monitor the kinetics of light adaptation, we tested the retinal response to a test flash of constant intensity superimposed on a light step. Two 20 ms flashes (9.6 μJ/cm^2^) were first delivered in the dark followed by the presentation of continuous background illumination (121 μJ/cm^2^ s) and test flashes repeated at 30 s intervals. The maximum amplitude recorded in a given data set was assigned the value 1. The time course of the incremental flash response, *bpeak(t)*, was fitted by a first order exponential of the form bpeak(t)=(DAbpeak-bpeak(t_0_))exp ((t+dly)/τ), where **_DA_bpeak is the peak amplitude of the dark adapted response, *bpeak*(*t *_0_ ) is the value of the exponential at *t *= *dly*, *dly *is the delay and τ is the time constant of the exponential.

### QUANTITATIVE REAL-TIME PCR

Total RNA from 7 dpf larval zebrafish (five larvae were pooled for each data point) treated with 0, 10^-^^7^, and 10^-^^6^ M dexamethasone for 24 h was extracted using TRIzol. cDNA was synthesized from total RNA with Superscript III Reverse Transcriptase (Invitrogen/Life Technologies, Carlsbad, CA, USA) using random hexamer primers (Amersham Biosciences, Piscataway, NJ, USA). Quantitative real-time PCR (qPCR) was performed using the SYBR Green PCR Master Mix kit (Applied Biosystems, Foster City, CA, USA) with gene-specific primer pairs that generated single PCR products. Data obtained from the PCR reaction were analyzed using the comparative CT method (Perkin Elmer Life Sciences, Waltham, MA, USA). The *cyclophilin A* gene was used as a control for normalization. PCR primer sequences: cyclophilin A (AY391451); forward TCACACTGAAACACGGAGGCA; reverse GCTTACCGTCCAGCCAGTTG. POMC (pro-opiomelanocortin; NM_181438); forward TGTCGAGACCTCAGCACAGA; reverse CTCGGAGGGAGGCTGTAGAT. Opsin (green opsin, RH-2; AB087805); forward GCTGT TCCAGCCTTCTTCTC; reverse TGTTAAGCATGCAGCTACGG. Transducin (Gnat2; NM_131869); forward GCATCTGCTTCCCTGACTATG; reverse TCCCTTCTTCATGTTCAGGTCT.

Tyrosine hydroxylase (TH; BX511171); forward AGGACGCCAAACAGAAGTTG; reverse GCTGCAAGTGTAGGGGTCAT. Dopamine D1 receptor (putative on chromosome 14; AL935195); forward CTGACCGGTTGCTTTCTTTC; reverse GGCTTGGTGTCTCCATGCTA.

Dopamine D1A3 receptor (XM_691439); forward TTGTAGGTTTTTGGCCGTTC; reverse GGATTTGTGCTGTCCGTTTT. Dopamine D2B receptor (AY333792); forward CACTCAGATGCTGGCTATCG; reverse TCACATATCCCAGCCAAGTG.

Dopamine D1C receptor (XM_686933); forward TTTCTCTGGCCGTTTCAGAT; reverse GTCCAGTCGAGGTGAATGGT. Dopamine D1X receptor (XM_690792); forward CTGTGCGTAATCAGCTTGGA; reverse TCCACGTTACGCTGACCATA.

Dopamine D4A receptor (AY750152); forward GGGTGCTTCCGGTTGTAGTA; reverse ACCCAACCAGGTCACGATAC. Gs alpha subunit (BX927257); forward AATACAACGCCTGGAAATGG; reverse CAAACATGCCAAAGCATACG.

### *IN SITU* HYBRIDIZATION

Zebrafish larvae were fixed in 4% paraformaldehyde (PFA) and subjected to staining by *in situ* hybridization. Full length coding sequence of the GR cDNA was used for the synthesis of the RNA probe (Promega, Fitchburg, WI, USA). After staining, the sample was sectioned by a vibratome to observe GR expression in the eye.

### IMMUNOHISTOCHEMISTRY

Zebrafish larvae at 7 dpf were fixed in 4% PFA and cryostat sections were subjected to fluorescent immunostaining. The primary antibodies used were MAB302a (Chemicon) for glutamine synthetase, MAB318 (Chemicon) for TH, and anti-arrestin antibody (SCT1-128) kindly provided by Dr. Paul Hargrave.

## RESULTS

### RECOVERY OF THE OPTOKINETIC RESPONSE IN *gr*^*s*^*357* MUTANTS IS DELAYED ON DARK/LIGHT TRANSITION

**FIGURE 1:**
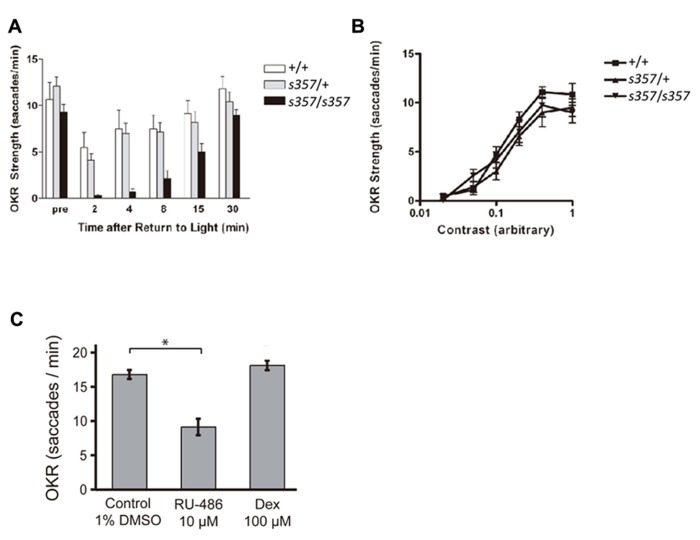
**Optokinetic responses are affected by disruption of glucocorticoid signaling. (A)** OKR, in saccades per minute, to motion of a constant sine-wave grating at high contrast. Zebrafish (7 dpf) were initially light adapted (pre) and measured at the indicated time points following a 45 min dark period. *gr *^*s357*^ mutants (*n *= 7; confirmed by genotyping) recovered more slowly from the dark than either WT (+/+; *n *= 6) or heterozygotes (*s357*/+; *n *= 15). Asterisks: significant difference (*p *< 0.05). Bars: Standard error of the mean (SEM). **(B)** Contrast sensitivity of OKR. Light-adapted mutants (*n *= 12) were not different from heterozygotes (*n *=**10) or WT (*n *=**13). Bars: SEM. **(C)** Changes in OKR strength of WT fish (7 dpf) following pharmacological reduction or elevation of GR activity. Overnight treatment with RU-486 (RU, 10 μM) diminished the number of saccades to a constant stimulus, whereas overnight dexamethasone (Dex, 100 μM) barely increased them. Control treatment was 1% DMSO. Bars: SEM. Asterisk: significant difference (*p *< 0.001).

The GR mutant allele, *gr*^*s357*^ (originally named *utouto *^*s357*^ ) was isolated in a large-scale screen for ethylnitrosourea-induced mutations disrupting visual behavior (Muto et al., 2005). Positional cloning was used to identify the mutated locus. By sequencing GR cDNA from wild type and mutants, a missense mutation was identified (arginine at position 443 to cysteine in GR; Ziv et al., 2012). To assess visual function in the *gr *^*s357*^ mutant, we employed the OKR assay, which measures the fishes’ reflexive eye movements to a large-field motion stimulus (Brockerhoff et al., 1995; Neuhauss et al., 1999). At 6 and 7 dpf, the larval stages tested here, visual responses are driven exclusively by cone photoreceptors; rods are not yet functional (Bilotta et al., 2001). When adapted to the ambient light in the recording setup, *gr *^*s357*^ homozygous larvae showed a normal OKR, which was comparable to homozygous WT and heterozygous siblings in amplitude of eye movements and frequency of saccades (**Figure 1A**; compare pre-dark responses). The OKR of the light-adapted mutant to varying contrasts was also similar to WT (**Figure 1B**). These results indicate that visual function in the mutant fish is normal under steady-state conditions.

Differences between mutant and WT became apparent when the visual system was challenged with a sudden transition from dark to light. After a period of darkness (45 min or more), the OKR of homozygous WT and heterozygotes was initially suppressed; the eye movements showed fewer saccades per time, while retaining their amplitude. Previous work had established that saccade rate is closely correlated with perceived contrast of the moving stimulus. When visual sensitivity is compromised or when the stimulus is less salient, fewer saccades are observed, whereas eye movement amplitudes do not change (Roeser and Baier, 2003; Rinner et al., 2005). The WT OKR fully recovered within a few minutes in response to constant light. In contrast, the OKR of homozygous *gr *^*s357*^ mutants took >30 min of light exposure to reach pre-dark levels (**Figure 1A**). Together, this behavioral phenotype is consistent with a defect in retinal light adaptation (Page-McCaw et al., 2004).

### THE LIGHT ADAPTATION DEFECT IN *gr*^s^357 MUTANTS IS LIKELY CAUSED BY TRANSCRIPTIONAL CHANGES

Next we asked if the effect on visual adaptation is caused by transcriptional (i.e., slow, long-term) or non-genomic (i.e., acute) effects of altered GR function (Groeneweg et al., 2012). Overnight bath application of the GR antagonist RU-486 (10 μM; overnight) reduced the WT OKR (in number of saccades per minute) to a constant grating stimulus (**Figure 1C**; *p *< 0.001; two-tailed Student’s *t*-test). Dexamethasone (100 μM) did not show a significant effect (**Figure 1C**; *p *= 0.19 for the experiment shown). Short drug treatments (30 min) had no effect, even at high concentrations of RU-486 (20 μM; data not shown). The slow time course of pharmacological actions is consonant with the interpretation that GR signaling positively controls visual performance via transcriptional action.

### THE *gr*^*s*^357 MUTATION REDUCES PHOTORESPONSES OF THE DARK-ADAPTED RETINA

**FIGURE 2:**
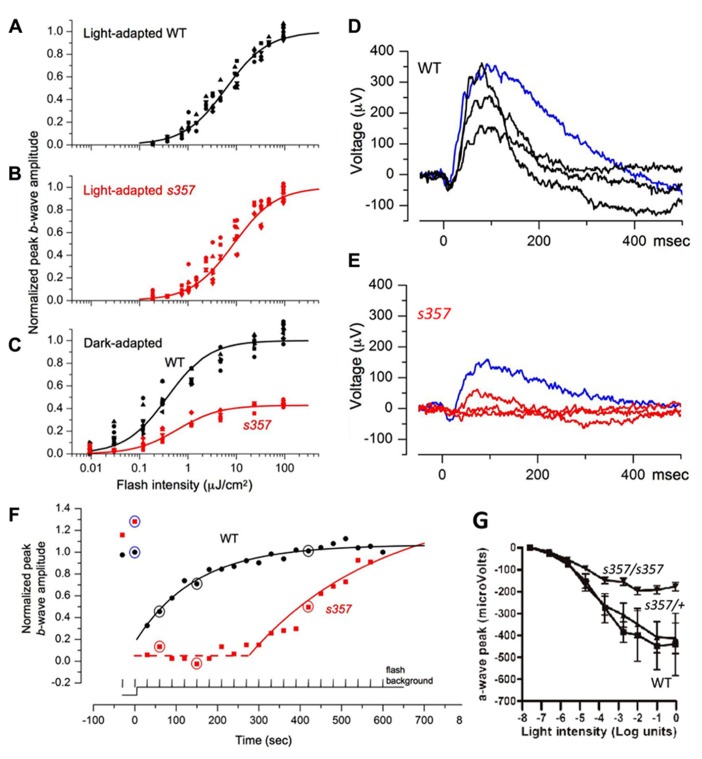
**Electrophysiological evidence for visual defects in *gr*^s357^ mutants. (A–C)** Normalized light sensitivity plots of light-adapted WT (**A**; *n *= 9) and mutant (**B**; *n *= 11) larvae, and dark-adapted WT (*n *= 8) and mutant (*n *= 5) larvae **(C)** at 7 dpf, fitted by the Naka-Rushton function (see Materials and Methods). Normalized peak amplitudes in the dark-adapted mutants were further normalized to wild type **(C)**. **(D–F)** Representative incremental flash ERG traces from WT **(D)** and mutant **(E)** larvae. Dark-adapted larvae were presented two 20 ms test flashes (blue traces; 9.6 μJ/cm^2^, nearly saturated peak amplitude) prior to the onset of continuous background illumination (121 μJ/cm^2^ s) followed by 20 test flashes at 30 s intervals. WT (**D**, black) and mutant (**E**, red) traces are ERG signals generated by flashes delivered 60, 150, and 420 s after background onset. The dashed line indicates the delay in the onset of recovery in the mutant. **(G)** Reduction of *a*-wave amplitudes in dark-adapted *gr *^*s357*^ mutants. In these experiments, the *b*-waves were pharmacologically suppressed by bathing the fish in L-APB (1 mM, pH 7) for 60 min before recording. Peak photocurrents were reduced in the mutant (*n *= 9) at light intensities greater than log unit = -4.7. Threshold (at log unit = -5.64) of the photoresponse was not affected. Sibling larvae (*n *= 14; 9 heterozygotes and 5 homozygous WT) did not show significant differences over the light intensities tested. Bars: SEM. Light intensity was 81 mW/cm^2^ at log unit = 0.

To localize the light adaptation defect revealed in the OKR assay, we recorded ERGs. Flash ERGs demonstrated the typical cornea-positive peak (*b*-wave), which reflects bipolar cell activity. The *b*-wave is often preceded by a small cornea-negative *a*-wave, which corresponds to photoreceptor currents. In the light-adapted state, WT and mutant larvae showed no significant differences in both their maximal *b*-wave response (*bpeak*_max_ = 543 ± 250 μV for *n *= 9 WT vs. 485 ± 245 μV for *n *= 11 mutants) and in the light intensity that elicited a half-maximal response (σ = 6.15 ± 1.86 μJ/cm^2^ for WT vs. 8.82 ± 4.95 μJ/cm^2^ for mutants; mean ± SD; **Figures 2A,B**). However, the ERG responses of mutants that were thoroughly dark-adapted (>3 h) were substantially smaller than those of WT. In half of the dark-adapted mutants, responses could not be elicited at all over the range of intensities tested. In the remaining mutants, the maximum amplitude was reduced (*bpeak*_max_ = 438 ± 192 μV for *n *= 8 WT vs. 188 ± 56 μV for *n *= 5 mutants; two tailed *t*-test, *p *≤ 0.004; **Figure 2C**). The dark-adapted ERGs showed the expected shift in the mean value of σ (3.74 ± 1.65 μJ/cm^2^ for WT and 6.04 ± 2.76 μJ/cm^2^ for mutants) and were not significantly different. Response thresholds of the dark-adapted mutant retina, when measurable, were unchanged (**Figure 2C**). ERG recordings of isolated *a*-waves corresponding to photoreceptor light responses, after treatment of the dark-adapted retina with L-2-amino-4-phosphonobutyrate (L-APB), showed that phototransduction currents were on average smaller in the mutant (**Figure 2G**). Together, these findings argue that threshold sensitivity is normal in the steady state, but that light responses are attenuated in the mutants.

We next asked if our ERG protocol could reveal differences during the process of light adaptation. Mutant and WT fish were initially dark-adapted. Then, test flashes of constant intensity were superimposed on a continuous light step and repeated at 30 s intervals (see Materials and Methods). In WT, the first flash delivered after background onset generated a smaller response than the dark-adapted one. Over time, the retina responded to the repeated flash with progressively larger amplitudes (**Figures 2D,F**). In mutants, continuous illumination entirely suppressed the flash response for several minutes, followed by a slow recovery of the flash response, until response saturation was attained (**Figures 2E,F**). This time course was well described by a first-order exponential with a delayed onset (*dly*) and a time constant (τ). Both parameters were statistically different between mutants and WT (*dly *= 3.2 ± 8.8 s vs. 411 ± 116 s,* p *<**10^-^^5^; τ = 183 ± 56 s vs. 265 ± 57 s,* p* < 0.005, for 8 WT and 5 mutants, two tailed *t*-test). Thus, the sluggish recovery of the mutant’s retinal responses is the likely cause of its failure to perform OKR behavior after a similar transition from dark to light.

### CANDIDATE GENES INVOLVED IN VISUAL PHYSIOLOGY ARE REGULATED BY GR TRANSCRIPTIONAL ACTIVITY

**FIGURE 3:**
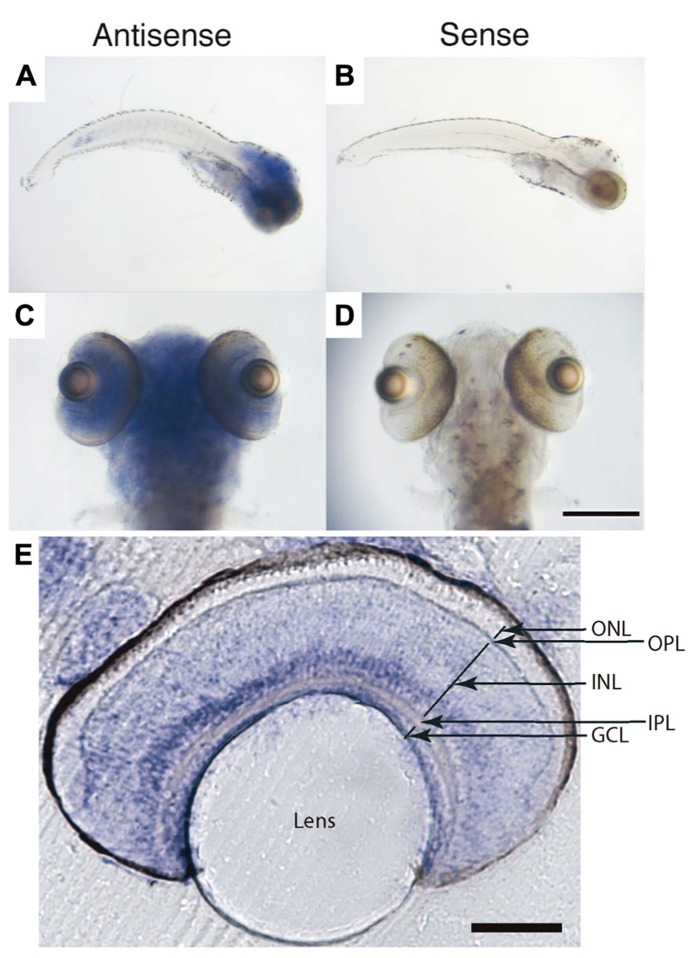
**Glucocorticoid receptor mRNA expression in zebrafish larvae at 7 dpf. (A–D)** Whole-mount *in situ* hybridization. Antisense labeling **(A, C)** showed GR expression in the entire brain including the retina, and also in internal organs. Sense controls **(B, D)** showed no signal. **(E)** Vibratome section of the retina following whole-mount staining. Strong signals were observed in the inner nuclear layer and ganglion cell layer. ONL, outer nuclear layer (photoreceptor layer); OPL, outer plexiform layer; INL, inner nuclear layer; IPL, inner plexiform layer; GCL, retinal ganglion cell layer. Scale bar: 350 μm in **(C, D)**, 100 μm in **(E)**.

**FIGURE 4:**
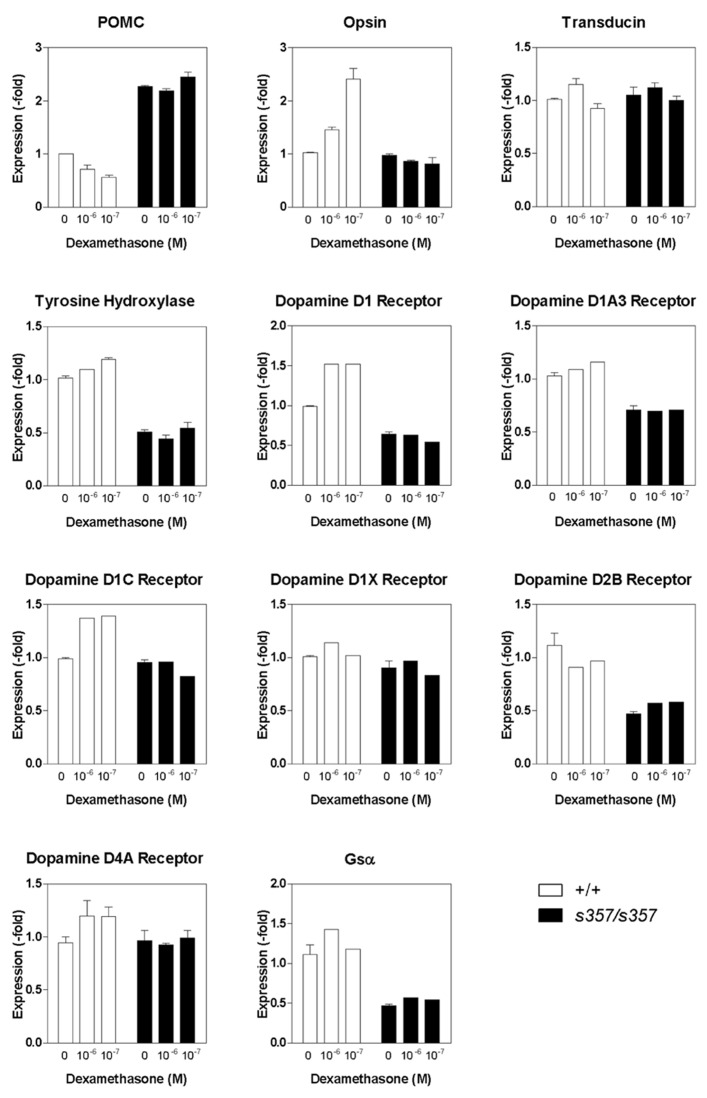
**Expression of vision-related genes in the *gr*^s357^ mutant as determined by real-time quantitative PCR.** Dexamethasone (10^-^^6^ and 10^-^^7^ M) was bath-applied to 6 dpf fish. Vehicle (ethanol) was used as control. RNA was isolated 24 h later. Cyclophilin A was used as a control to which other gene expression levels were normalized.

Glucocorticoid receptor is expressed in most cells of the larval zebrafish retina, predominantly in the inner nuclear and ganglion cell layers, as determined by RNA *in situ* hybridization (**Figure 3**). An important neuromodulator that is known to function in the light adaptation process is dopamine (Dearry and Burnside, 1989). To reveal possible target genes of GR, we examined genes involved in dopaminergic signaling, as well as some other vision-related genes. We measured the expression levels of 11 candidate genes by quantitative RT-PCR, either untreated or following a 24-h treatment with dexamethasone (0.1 and 1 μM). From this small sample, we found at least half of the genes to be misregulated, among them a randomly chosen cone opsin (RH2, expressed in M cones) and a number of dopamine signaling components (**Figure 4**). The expression of TH, D1 and D2 receptor isoforms, and G_s_α were decreased in the mutant. The expression of these genes was not responsive to dexamethasone, as partially predicted by the presence of glucocorticoid response elements in their promoters (Zhou et al., 1992; Hagerty et al., 2001; **Figure 4**). The transcription of the gene encoding the ACTH precursor POMC was increased in *gr *^*s357*^ , as expected from the presence of negative control sequences in the POMC promoter (Drouin et al., 1993; Martens et al., 2005). From the large fraction of misregulated genes in this small sample, we conclude that GR likely controls an extensive array of target genes in the retina.

**FIGURE 5:**
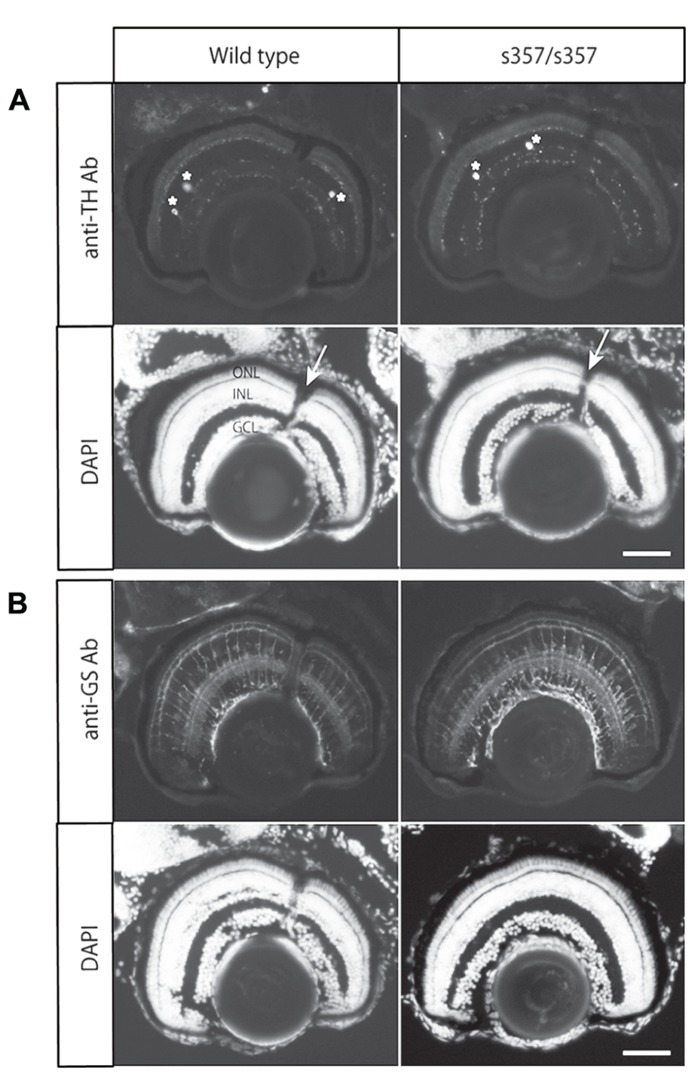
**Immunohistochemical analysis of TH and glutamine synthetase in the retina. (A)** Immunostaining with a monoclonal antibody (Ab) against TH. Cell bodies of the TH-positive cells in the section are shown with asterisks. Arrows in nuclear staininig (DAPI) shows the optic nerve. **(B)** Immunostaining with anti-glutamine synthetase (GS) antibody. Note that there were no significant difference in the staining intensity and patterns between wild type and the mutant retina. ONL, outer nuclear layer (photoreceptor cell layer); INL, inner nuclear layer; GCL, retinal ganglion cell layer. Scale bar: 50 μm.

**FIGURE 6:**
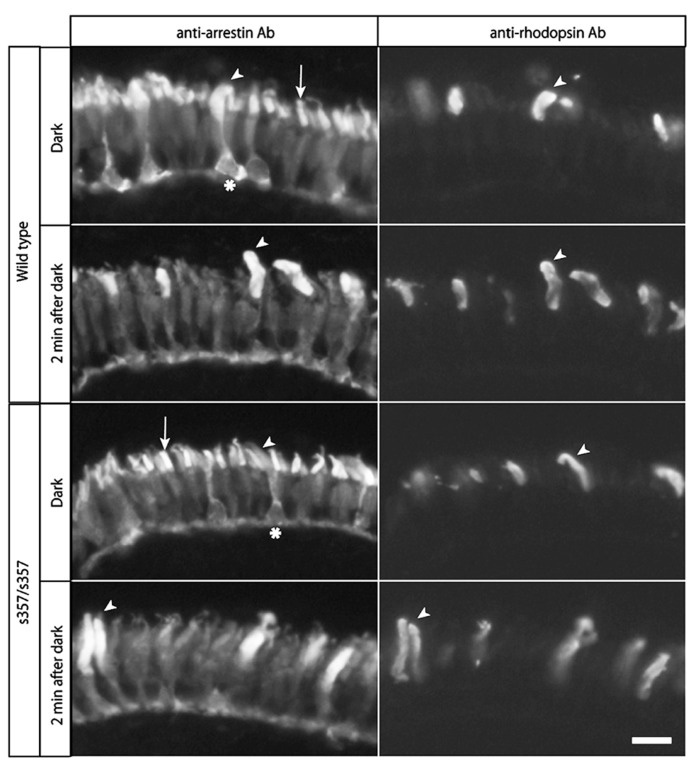
**Normal arrestin translocation in the retina.** Larval retinae were subjected to double-immunostaining with anti-arrestin monoclonal antibody (left panels) and anti-rhodopsin antibody (right panels). Arrestins in both cone photoreceptors (arrows) and rod photoreceptors (arrow heads) were recognized by this antibody. In rod photoreceptor cells, arrestins were distributed across the entire cell (asterisks indicate rod cell bodies), and accumulated in the outer segments in light (2 min after 45 min dark treatment). In cone photoreceptors, arrestins were present predominantly in the outer segments in dark, and were more broadly distributed in light. Note that there were no significant differences in the light-dependent arrestin localization between the wild type and mutant retina. Scale bar: 10 μm.

As developmental or cellular-level defects could explain the adaptation defect in *gr *^*s357*^ , we examined retinal sections by immunohistochemistry. The number of dopaminergic interneurons (TH-positive cells) and glutamine synthetase-expressing Müller glia were unchanged (**Figure 5**). Light-induced translocation of arrestin to the rod outer segment, a photoreceptor-autonomous light-adaptive process (Calvert et al., 2006), also occurred normally in the mutant (**Figure 6**). We conclude that visual adaptation defect in in *gr *^*s357*^ is most likely caused by misregulated vision-related gene expression, but not due to altered retinal cell differentiation or photoreceptor cellular architecture.

## DISCUSSION

By analyzing the zebrafish *gr*^*s357*^ mutant, we discovered a role for GR in the regulation of retinal light adaptation. A delay in light adaptation causes defects in visual behavior (OKRs) during dark/light transition, the phenotype by which the mutant was originally identified (Muto et al., 2005). Glucocorticoids, like other steroid hormones, have both genomic and non*-*genomic mechanisms of actions. Based on the slow time course of pharmacological treatments with agonists and antagonists of GR, we suggest that transcriptional effects underlie the sluggish light adaptation seen in our mutant. We excluded developmental defects as a cause of the phenotype as the mutant retina did not show any morphological abnormalities in the cell types examined (**Figures 5 and 6**; Muto et al., 2005). Taken together, our data indicate that glucocorticoids regulate retinal adaptation via transcriptional control of vision-related genes.

The *gr*^*s357*^ mutation eliminates DNA binding, thus both transactivation and transrepression of target genes are impaired (Ziv et al., 2012). What are the candidate genes whose misregulation in *grs357 *might delay light adaptation? We predicted that genes involved in dopamine signaling might play a role in this process, because dopamine is a key neuromodulator of the retinal network (Vaquero et al., 2001; Witkovsky, 2004), and its deficiency results in visual-system phenotypes in zebrafish reminiscent of those seen in the *grs357* mutant (Li and Dowling, 2000). Indeed, our results suggest that at least some of the genes in dopamine signaling are affected by *gr *^*s357*^ mutation. In our qPCR experiments, we observed a moderate level of dexamethasone-induced changes in gene expression (**Figure 4**). One reason for this could be that glucocorticoid-mediated gene expression is differentially regulated in different tissues and that using whole embryos may underestimate changes specific to the retina. Considering that there are hundreds of possible GR target genes (Phuc Le et al., 2005), it is likely that GR regulates an extensive gene network whose cumulative effect enables rapid light adaptation.

Whereas light perception is well known to modulate neuroendocrine functions, including stress, sleep, and immune responses (Roberts, 2000), our study is the first to identify the reverse relationship, the control of visual physiology by glucocorticoid hormones. This regulatory loop could potentially serve two complementary functions. First, glucocorticoids may provide a direct, light-driven feedback signal to the retinal circuitry, adjusting visual physiology to ambient light levels, complementing dopamine and other neuromodulators. Second, glucocorticoids could integrate the HPA axis with the sensory periphery, as part of a whole-organism coping mechanism that facilitates adjustment to rapid environmental changes (Pecoraro et al., 2006).

## Conflict of Interest Statement

The authors declare that the research was conducted in the absence of any commercial or financial relationships that could be construed as a potential conflict of interest.

## AUTHOR CONTRIBUTIONS

Akira Muto and Herwig Baier designed experiments and wrote the manuscript. Akira Muto, Michael R. Taylor, Miyuki Suzawa, and Juan I. Korenbrot designed and performed experiments and analyzed the data.
